# Relearning the lesson – amelanotic malignant melanoma: a case report

**DOI:** 10.1186/1752-1947-2-31

**Published:** 2008-01-31

**Authors:** Ezekiel Oburu, Alberto Gregori

**Affiliations:** 1 Department of Orthopaedics, Hairmyres Hospital, East Kilbride, G75 8RG, UK

## Abstract

Although not as common as the other melanomas, amelanotic melanoma often evades diagnosis by masquerading as other pathology. A high index of suspicion is therefore required for early and appropriate intervention. We present a patient who was diagnosed and managed as having paronychia of the middle finger while in actual fact he had a subungual amelanotic melanoma. By the time of his referral to the orthopaedic team it had progressed to an advanced stage. Our case underlies the importance of early recognition and referral of this rare but malignant lesion by primary care physicians.

## Background

With malignant melanoma early diagnosis is vital. Amelanotic malignant melanoma often presents in unusual ways, often evading early diagnosis, resulting in a poorer prognosis. Differential diagnosis can include paronychia, pyogenic granuloma, glomus tumor, and subungual haematoma. Our case highlights that any persistent ulcer adjacent or below the nail not responsive to treatment should raise suspicion.

## Case presentation

**Figure 1:**
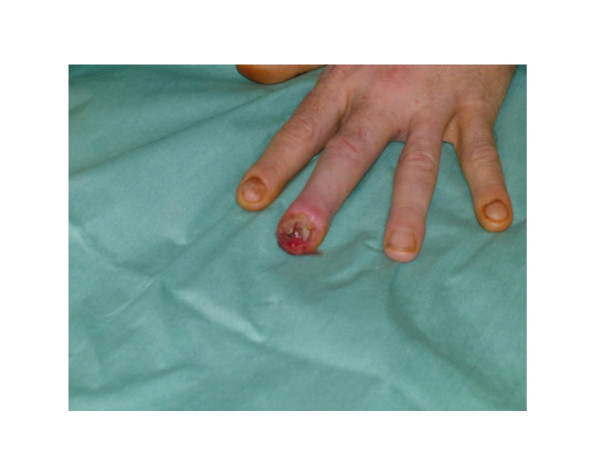


A 55 year old male presented with a 10 month old painless ulcer of the left middle finger (Figure 1). Being a nail biter the initial diagnosis was paronychia having discharged pus. Nail removal was attempted and antibiotics were administered. The wound was subsequently dressed for months without improvement.

Examination revealed an ulcerated swollen fingertip with partial nail loss.

**Figure 2:**
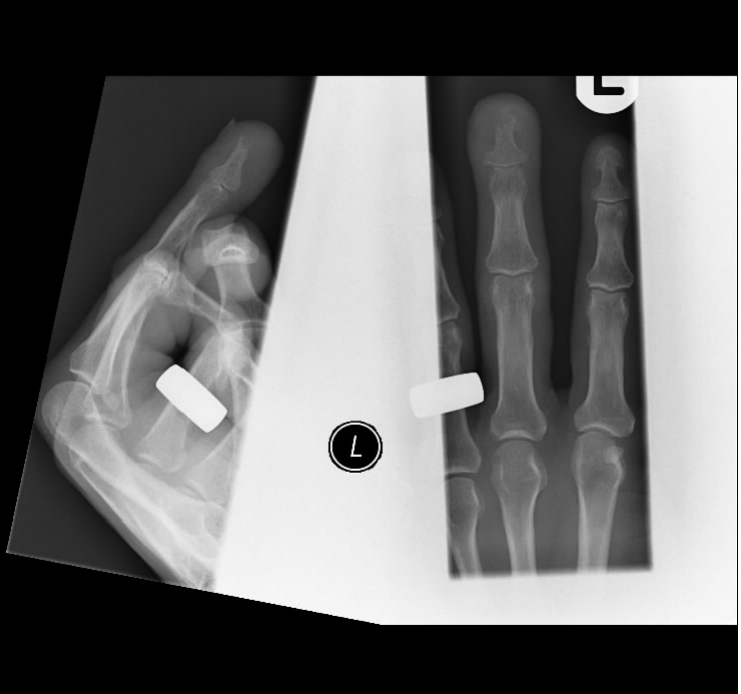


Lymphadenopathy was not clinically evident. Haematological parameters were normal. Radiology revealed a distal phalangeal radiolucent lesion (Figure 2). An excision biopsy diagnosed amelanotic melanoma with a Breslow level of 6 mm. The patient later developed pulmonary metastasis and died.

## Discussion

Melanoma not only presents to dermatologists, but to other medical practitioners and early diagnosis is vital. Patients discover approximately half of melanomas, a quarter are detected by medical providers [1]. Amelanotic melanomas comprise only 2% of melanomas [2] and is most commonly subungual [3].

Prognosis is dependant on the Breslows level at time of diagnosis. In amelatonic melanoma the cues leading to diagnosis are often absent, leading to reports of missed diagnoses and poorer prognoses. Evaluating this patient's presentation suggests that an earlier diagnosis was possible. Nail loss can occur in subungual melanoma and lesions affecting the nail bed associated with nail plate lifting are suspicious [4]. Lack of ulcer healing is another sign suggestive of underlying malignancy. The radiological appearance was also suggestive of malignancy. Elmets [5] reported a sixty-two year old man with a right hallux amelanotic melanoma diagnosed after the lesion had been treated for months as a pyogenic granuloma. Establishment of the correct diagnosis was aided by finding a radiolucent defect on radiology.

Underlying bone involvement and a Breslow level of 6 mm is confirmation of a late diagnosis. In reviewing 24 patients with subungual melanoma Rigby [6] found a mean diagnostic delay of 30 months. The timing of diagnosis is critical with better survival rates in cases of early diagnosis and treatment.

Non healing ulcers distorting the digital nail bed should engender a high index of suspicion of malignancy and demand radiology and early biopsy [7].

## Conclusion

This case report emphasizes the importance of early diagnosis of amelanotic melanoma and the need for a high index of suspicion on the part of the primary care physician. Non healing ulcers adjacent to the nail bed should be investigated by early biopsy and radiology.

## Competing interests

The author(s) declare that they have no competing interests.

## Authors' contributions

EO (SHO) managed the patient and wrote the report. AG (Consultant) performed surgery and supervised and edited the report. Both authors read and approved the final manuscript.
